# A Review of Healthy and Fibrotic Myocardium Microstructure Modeling and Corresponding Intracardiac Electrograms

**DOI:** 10.3389/fphys.2022.908069

**Published:** 2022-05-10

**Authors:** Jorge Sánchez, Axel Loewe

**Affiliations:** Institute of Biomedical Engineering, Karlsruhe Institute of Technology (KIT), Karlsruhe, Germany

**Keywords:** cardiac modeling, fibrosis, electrogram, multiscale, microstructure

## Abstract

Computational simulations of cardiac electrophysiology provide detailed information on the depolarization phenomena at different spatial and temporal scales. With the development of new hardware and software, *in silico* experiments have gained more importance in cardiac electrophysiology research. For plane waves in healthy tissue, *in vivo* and *in silico* electrograms at the surface of the tissue demonstrate symmetric morphology and high peak-to-peak amplitude. Simulations provided insight into the factors that alter the morphology and amplitude of the electrograms. The situation is more complex in remodeled tissue with fibrotic infiltrations. Clinically, different changes including fractionation of the signal, extended duration and reduced amplitude have been described. *In silico*, numerous approaches have been proposed to represent the pathological changes on different spatial and functional scales. Different modeling approaches can reproduce distinct subsets of the clinically observed electrogram phenomena. This review provides an overview of how different modeling approaches to incorporate fibrotic and structural remodeling affect the electrogram and highlights open challenges to be addressed in future research.

## 1 Introduction

Patients with cardiac arrhythmias are often treated with ablation therapy. Substrate-based ablation therapy is guided by intracardiac measurements acquired from catheters inserted into the cardiac chamber that record the extracellular potential.

The signal recorded by an electrode with respect to a distant reference is called unipolar electrogram (uEGM). EGMs of several electrodes on a catheter and/or multiple catheter locations are used to understand the dynamics of the cardiac arrhythmia. However, the recorded uEGMs are affected by different artifacts such as contraction of the heart, breathing of the patient, far-field signals from distant parts of the heart and noise from different hardware components. To alleviate these issues, bipolar electrograms (biEGM) are most frequently used, which subtract the uEGMs of two close-by electrodes. In this way, artifacts that affect both electrodes in the same way are cancelled. The difference between two potentials is called voltage and we should keep in mind that we can only measure voltages. Therefore, uEGMs always have to be considered with respect to their (distant) reference electrode. In clinical literature, also the peak-to-peak amplitude of an electrogram signal (i.e., a voltage time course) is often called “voltage”.

The mathematical model of an excitable cell proposed by Hodgkin and Huxley, (1952), the tissue homogenization approach proposed by Schmitt, (1969), and the set of bidomain equations first applied by Tung, (1978) in 1978 is the most complete and accurate model that describes the spread of electrical depolarization across the myocardium and its cells.

Computational simulations based on this mathematical model have been used to understand the phenomena of the depolarization spread in cardiac tissue and their effects on electrogram genesis and morphology (Bishop and Plank, 2011b; Oesterlein et al., 2016; Roney et al., 2016; Pollnow et al., 2017; Beheshti et al., 2018; Hwang et al., 2019). While EGMs can be extracted from the extracellular medium in a bidomain simulation, this approach is computationally expensive. Thus, different methods based on excitation propagation simulations in the monodomain model have been proposed. Another modeling approach to accelerate the computation is the so-called reaction-eikonal model Neic et al. (2017), which can simulate physiological propagation using a coarser mesh (element average length 400 μm). In the monodomain model and the reaction-eikonal model, the extracellular potential is not calculated directly. However, it can be approximated with the pseudo-bidomain approach or the infinite homogeneous volume conductor method to obtain EGMs as detailed below. The infinite homogeneous volume conductor method approximates the extracellular potential caused by a group of cells spatially distributed in space and acting as sources of the electric field (Malmivuo and Plonsey, 1995).

In this review, we give an overview of the biophysical phenomena governing wave propagation in cardiac tissue and the corresponding extracellular potentials measured as electrograms. We will particularly focus on different approaches used to model fibrotic remodelling and simulate the corresponding electrograms to reproduce and understand the clinically observed changes in electrogram amplitude and morphology.

## 2 Intracardiac Electrograms

The electrical activity in the myocardium originates from the coordinated opening and closing of the ion channels in the cell membrane. The time course of the difference between the potential in the intracellular and in the extracellular medium is known as the action potential. In cardiac tissue, the cells are interconnected through gap junctions that will start a cascade effect of cellular activation along the major axis in which myocytes are aligned locally (also known as fiber direction), resulting in excitation propagation across the myocardium.

**FIGURE 1:**
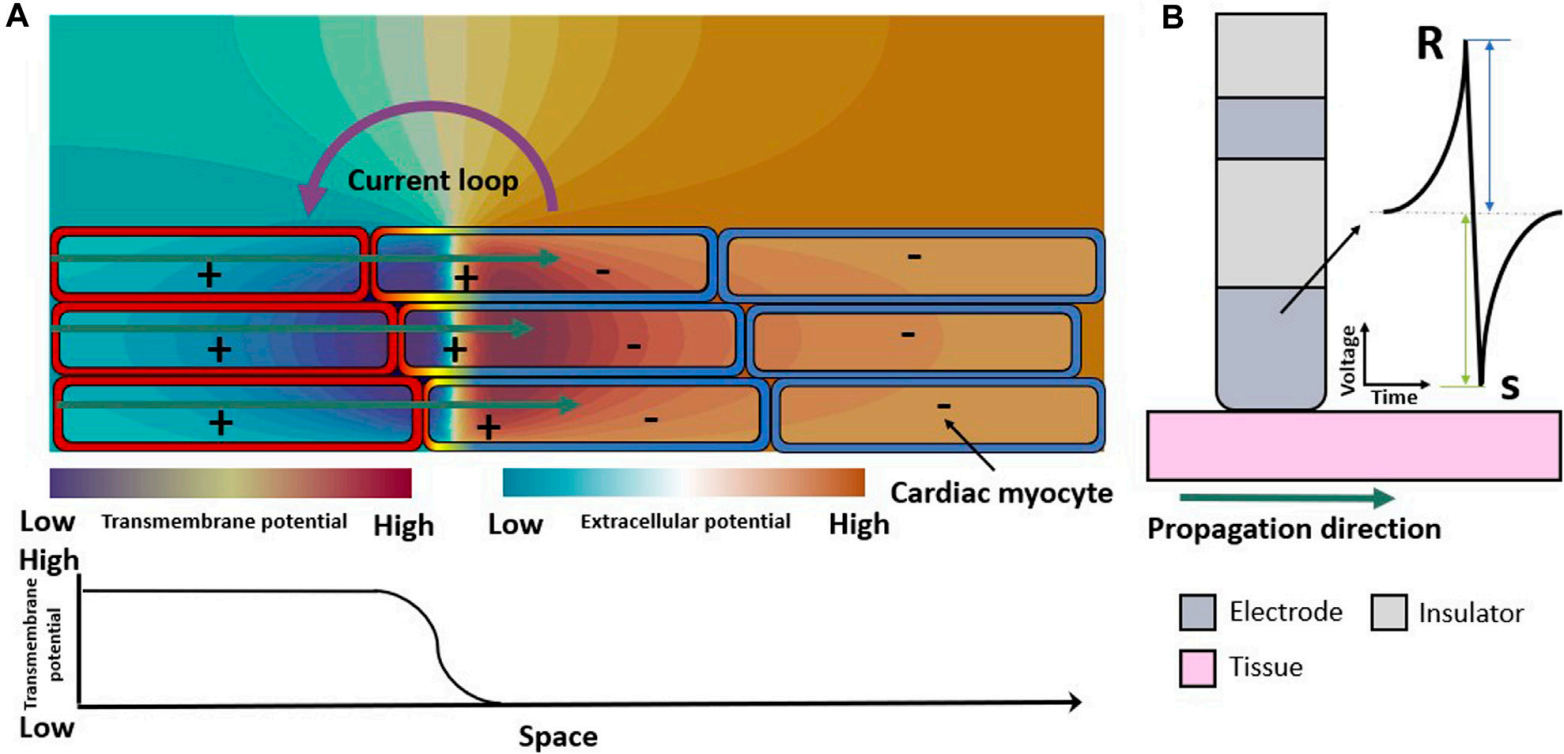
Electrical propagation in healthy cardiac tissue. **(A)** Extracellular field caused by the depolarization of cardiomyocytes when an excitation propagates from left to right (green arrows). Spatial transmembrane voltage distribution is color-coded on the membranes and shown in the bottom row. The leftmost cells are already depolarized an in the action potential plateau while those on the right are still at resting membrane voltage. **(B)** Symmetric unipolar electrogram measured at the surface of the cardiac tissue (pink). The initial positive wave (R-peak) is caused by the wavefront approaching the electrode (dark gray), the polarity changes when the wavefront passes underneath the electrode, and the S-peak is caused by the wavefront traveling away from the measuring electrode.

The extracellular field is a consequence of the spatial distribution of the transmembrane voltage of the cells in the myocardium (Figure 1A). An advancing depolarization wave in the cardiac tissue changes the spatial distribution of the extracellular potential. The extracellular potential can be measured as the uEGM at one electrode (technically the voltage between the extracellular potential at the measuring electrode with reference to for example, Wilson central terminal). The unipolar electrogram morphology is characterized by a biphasic symmetric shape (Figure 1B) where the positive phase (R-peak) indicates the approaching of the wavefront to the measuring electrode and the fast downslope indicates the moment that the wavefront is underneath the electrode. The opposing negative phase (S-peak) indicates the movement of the wavefront away from the measuring electrode. The peak-to-peak amplitude of the signal is also called “voltage” in the clinical literature. Peak-to-peak voltage is used as a marker to distinguish healthy from pathological tissue both for biEGMs (Jadidi et al., 2020) and uEGMs (Nairn et al., 2020b). However, biEGM amplitude can be affected by to several factors (Hwang et al., 2019) such as the orientation of the catheter (Schuler et al., 2013; Gaeta et al., 2020), the electrode spacing and size (Beheshti et al., 2018; Abdi et al., 2020; Nairn et al., 2020a; Takigawa et al., 2022), depolarization patterns (Jacquemet et al., 2003), substrate remodeling (Jacquemet et al., 2003; McDowell et al., 2012; Campos et al., 2013; Mendonca Costa et al., 2014; Roney et al., 2016; Sánchez et al., 2021b) and signal filter settings (Starreveld et al., 2020).

## 3 Modelling Intracardiac Signals

Computational cardiac modeling has advanced rapidly in the last years and different numerical methods to simulate the propagation of the cardiac depolarization have been proposed over the years. Finite difference approaches have been widely used (Potse et al., 2006) and can be generalized for grids with distinct spacing (Trew et al., 2005; Sánchez et al., 2019a). Also the finite element method has been used to discretize complex geometries such as the cardiac chambers to simulate cardiac electrophysiology (Vigmond et al., 2003; Cooper et al., 2015; Neic et al., 2017; Plank et al., 2021).

The bidomain model represents cardiac tissue as a homogenized medium composed of the intracellular and the extracellular domains. The two computational domains coexist in the bidomain model and occupy the same geometrical space:

$$\nabla \cdot \left.\left(\sigma_{i}\nabla \phi_{i}\right)\right)=\beta I_{m}$$ (1)

$$\nabla \cdot \left.\left(\sigma_{e}\nabla \phi_{e}\right)\right)=-\beta I_{m}-I_{\mathrm{extra}}$$ (2)

$$I_{m}=C_{m}\frac{\partial V_{m}}{\partial t}+I_{\mathrm{ion}}\left(V_{m},\nu \right)-I_{\mathrm{intra}}$$ (3)

$$V_{m}=\phi_{i}-\phi_{e}\;,$$ (4)

where *ϕ* represents the electrical potential, the indices *i* and *e* refer to the intracellular and extracellular spaces, respectively. *σ* is the conductivity tensor, *β* is the surface to volume ratio of the myocytes and *I*_*ion*_ the total transmembrane ionic current density defined by the cellular model. The latter is dependent on *V*_*m*_ and a vector *ν* of further state variables. *I*_*intra*_ (a transmembrane current density) and *I*_*extra*_ (an extracellular current density) describe external stimuli. If a bath surrounds the tissue, it is treated as an extension of the extracellular space.

Adding Eqs 1, 2 and incorporating it into Eq. 4 yields:

$$\nabla \cdot \left(\sigma_{i}+\sigma_{e}\right)\nabla \phi_{e}=-\nabla \cdot \left(\sigma_{i}\nabla V_{m}\right)-I_{\mathrm{extra}}$$ (5)

$$\nabla \cdot \left(\sigma_{i}\nabla V_{m}\right)=-\nabla \cdot \left(\sigma_{i}\nabla \phi_{e}\right)+\beta I_{m}\;.$$ (6)

As mentioned before, the reference potential during an electro-anatomical mapping procedure is usually a potential in a remote site or an average of potential values such as Wilson’s central terminal. For a bidomain model, when calculating uEGMs, the reference potential can, for example, be considered as an average of the extracellular potential of the furthest surface with respect to the tissue (Colli Franzone et al., 2007; Keller et al., 2014), which is not a perfect approximation of a remote reference electrode (e.g., a surface patch on the back of the patient) but markedly reduces drift of the reference potential. The further away the reference is from the myocardial tissue in the model, the better the representation of the reference potential but also the higher the computational cost due to the extended computational domain. Considering the average potential in a remote surface or volume is numerically advantageous compared to defining a fixed reference potential as a Dirichlet boundary condition.

The monodomain model is an approximation that assumes that the anisotropy of the extracellular and intracellular conductivity are aligned. Therefore, under the assumption of equal anisotropy ratios, one needs to solve only the parabolic partial differential equation above with the monodomain conductivity set appropriately:

$$\nabla \cdot \left(\sigma_{m}\nabla V_{m}\right)=\beta I_{m}+\beta I_{t}r\;,$$ (7)

where the bidomain equivalent monodomain conductivity *σ*_*m*_ is given as

$$\sigma_{m}=\sigma_{i}\sigma_{e}{\left(\sigma_{i}+\sigma_{e}\right)}^{-1}\;.$$ (8)

Potse et al. (2006) performed a thorough comparison between the results of the bidomain model and monodomain model. The authors conclude that the monodomain model, although being a simplification of the bidomain model, is sufficient to study and understand the electrical propagation in the cardiac tissue under physiological conditions as well as for electrically remodeled tissue (ionic current abnormalities). The acceleration of the wavefront at the tissue-to-blood interface due to the bath-loading effect can be represented with the augmented bidomain approach (Bishop and Plank, 2011b). One of the biggest disadvantages of the bidomain model is the long computation time that it requires. Therefore, a common modeling approach is to combine the monodomain model with independent forward calculation of extracellular potentials. The most simplistic approach is the infinite volume conductor assumption, which assumes that the cardiac tissue is immersed in a homogeneous extracellular medium with infinite extent. This approach was for example, used to study the relation of the spread of depolarization in the cardiac tissue to the genesis and morphology of the unipolar electrogram (Gima and Rudy, 2002; Ganesan et al., 2013; Ugarte et al., 2014; Cabrera-Lozoya et al., 2017; Hwang et al., 2019) but neglects the influence of the heterogeneous surrounding tissue like other cardiac chambers, the lungs or the liver.

Briefly, the source and the measuring point (electrode) for a dipole are assumed to be immersed in an unbounded (infinite) volume conductor with homogeneous properties. The time course of the potential of the dipole corresponds to the uEGM electrogram measured at a location *x* in a certain distance to the source located in the cardiac tissue (*x*_*src*_) with respect to a reference electrode in infinite distance using the integral solution to Poisson’s equation:

$$\phi_{e}=\frac{1}{4\pi \sigma }{∭}_{V}\frac{I_{\mathrm{src}}}{\left\|x-x_{\mathrm{src}}\right\|}\;dV\;,$$ (9)

where *ϕ*_*e*_ is the extracellular potential, *σ* is the conductivity of the volume conductor, *I*_*src*_ is the source current density and ‖*x* − *x*_*src*_‖ is the Euclidean distance from the source point to the measuring point.

Bishop and Plank (2011a) proposed a combined bidomain and monodomain model (pseudo-bidomain) to calculate the extracellular potential. The proposed pseudo-bidomain approach computes the elliptic bidomain equation for a given transmembrane voltage distribution only at the time instants for which the extracellular potential is sampled. This approach is suitable to reproduce extracellular signals [EGM (Keller et al., 2012) and ECG (Nagel et al., 2022)] for a finite surrounding conductive medium (bath, potentially inhomogeneous) and is computationally efficient.

### 3.1 Factors Affecting the Intracardiac Signals

Using the bidomain model and realistic geometries of commercially available catheters can help to better understand EGM morphology (Schuler et al., 2013; Pollnow et al., 2017; Sánchez et al., 2021a). Schuler et al. (2013) modeled a realistic 7F catheter with two electrodes such that the tip was at the center of the tissue and in direct contact with the tissue patch surface. The catheter angle was changed with respect to the surface of the tissue (elevation) and to the wavefront propagation direction (rotation). Additionally, the authors explored the impact of the tissue thickness and conduction velocity on biEGM amplitude and duration. One of their main findings was that catheter orientation greatly affects the height and ratio of the positive and negative bipolar signal amplitude, which can be traced back to changes in the proximal signal. Moreover, the authors pointed out that the substrate characteristics (thickness and conduction velocity) mainly affect the biEGM peak-to-peak amplitude.

**FIGURE 2:**
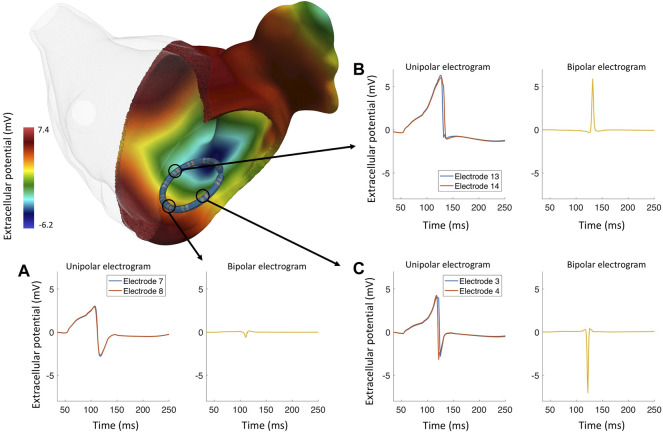
Bidomain simulation of a realistically deformed LASSO™ (Biosense Webster) catheter in a left atrium to study the genesis of different EGM morphologies in healthy myocardium. **(A)** The wavefront approaches the electrode pair 7–8 and activates both electrodes at the same time, the resulting bipolar electrogram with a reduced peak-to-peak amplitude (0.42 mV). **(B)** Several wavefronts approaching electrode pair 13–14, both unipolar electrograms are asymmetrical, lacking an S-peak; the resulting bipolar electrogram has a high peak-to-peak amplitude and a positive polarity. **(C)** The wavefront travels almost perpendicular to electrode pair 3–4; the electrodes are activated at different times, the resulting bipolar electrogram has negative polarity and a high peak-to-peak amplitude (7.45 mV).

In new highly detailed bidomain simulations for this review, we show the biophysical phenomena of the spread of depolarization in the left atrium and the EGMs from a 7F LASSO™ (Biosense Webster) catheter in a healthy left atrium. Figure 2 shows that local activation time is the main factor that impacts the biEGM amplitude and that it is less sensitive to the wavefront direction. Additionally, bidomain simulations showed that biEGMs from electrodes that are not in direct contact with the tissue have the same activation time resulting in a small biEGM amplitude, which confirms the results previously shown (Gaeta et al., 2020). In brief, the atrial anatomical model (Roney et al., 2021) has a realistic wall thickness and an average edge length of 100 μm. Tissue conductivity was tune to achieved a conduction velocity of 40 cm/s (McDowell et al., 2013). The value of conductivity of the blood were as reported by Clerc (1976), the electrode conductivity was set to 1 × 10^12^ S/m to represent a good conductor that yields an isopotential volume, the conductivity of the catheter insulator was set close to zero (1 × 10^6^ S/m).

The amplitude of uEGMs is affected by the geometrical properties of the electrode, such as the size of the electrode. Nairn et al. (2020a) performed a series of *in silico* experiments to understand the effect of the electrode size on the amplitude of the measured EGM. uEGM amplitude was shown to be inversely related to the size of the electrode. biEGM amplitude is additionally affected by the electrode pair spacing. Beheshti et al. (2018) showed that biEGM amplitude was increased when the electrode spacing increased. Assuming a plane wave and a perfectly symmetric uEGM in a simple thought experiment, the biEGM amplitude is zero for electrodes that are activated at exactly the same time. When increasing the distance between the electrodes, the peak-to-peak biEGM amplitude increases up to two times the uEGM amplitude. When further increasing the interelectrode distance, the biEGM amplitude decreases again until there is no more temporal overlap between the two uEGMs and the biEGM amplitude plateaus at the uEGM amplitude.

**FIGURE 3:**
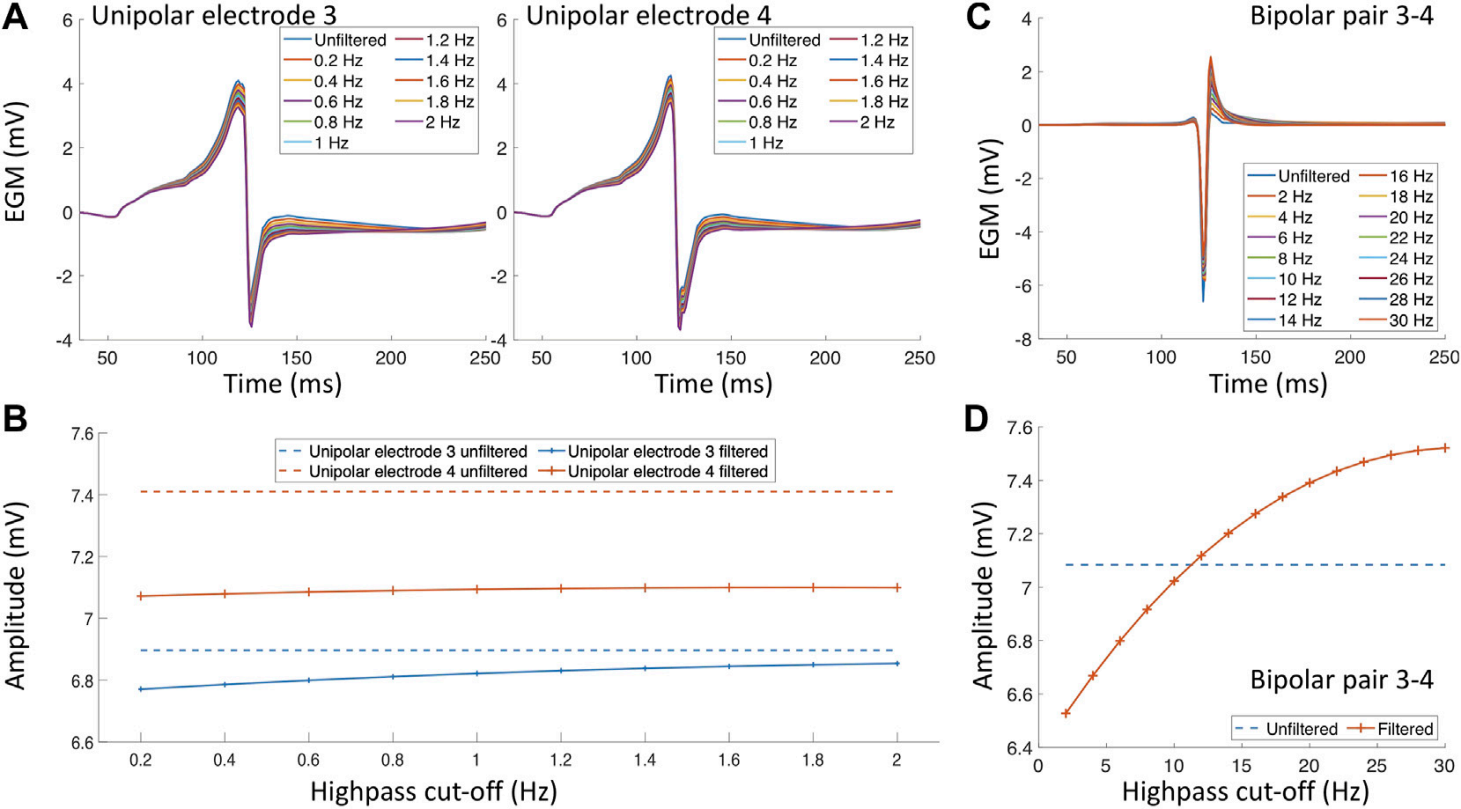
Filter effects on electrograms measured in healthy myocardium. **(A)** Unipolar electrograms with different highpass filter values. **(B)** Effect of different highpass cut-off values on the unipolar electrogram amplitude. **(C)** Bipolar electrogram with different highpass cut-off values. **(D)** Effect of different highpass cut-off values on the bipolar electrogram amplitude.

An additional factor that impacts the EGM amplitude and morphology are the filter settings (Schneider et al., 2004; Lin et al., 2007; Starreveld et al., 2020). In clinical practice, a bandpass filter is commonly used. However, the cut-off values of the bandpass filter differs for different mapping systems, catheters or due to the noise environment present in the specific electrophysiology laboratory. During an electroanatomical mapping procedure, uEGMs are typically filtered with a highpass of 0.5–2 Hz and a lowpass filter of 300–600 Hz biEGMs are typically bandpass filtered with a highpass of 1–30 Hz and a lowpass of 300–500 Hz. Both EGM types are also filtered at the frequency of the powerline with a notch filter (50 or 60 Hz). Figure 3 depicts the effect of the filter settings on both uEGMs (panel A) and biEGMs (panel C). In particular for biEGMs, the highpass filter cut-off value affects the measure voltage (Figure 3D). The higher amplitude of these simulated EGMs compared to clinical EGMs is likely due to the chosen extracellular conductivity, perfect contact of the electrode with the tissue and absence of losses along the signal chain.

Considering the numerous factors that affect the uEGM and biEGM amplitude and morphology, standardized mapping modality (uEGM or biEGM), electrode size, electrode spacing and filter settings could increase comparability between studies. For modeling the healthy myocardium and electrograms, bidomain models provide the most accurate representation of the biophysical phenomena of depolarization and the influence of the catheter inside the cardiac cavity. Monodomain models and reaction-eikonal models in combination with forward calculation approaches to obtain the EGMs provide sufficient information about the propagation in the cardiac tissue in most scenarios. After reviewing the factors that influence the EGMs in the healthy myocardium, the next section covers factors that increase the complexity of the signals due to heterogeneities of the tissue and different patterns of propagation.

## 4 Myocardial Structural Remodeling and Intracardiac Signals

**FIGURE 4:**
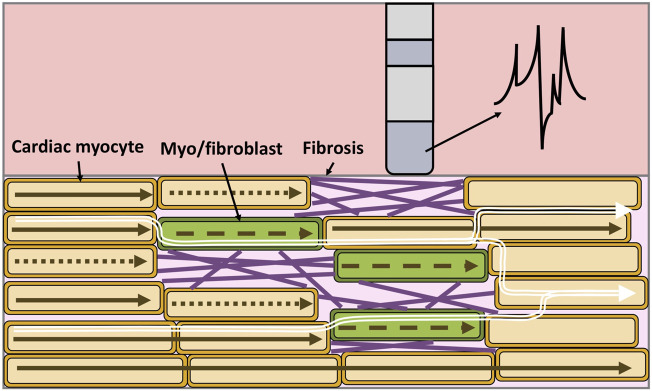
Electrical propagation in fibrotic cardiac tissue, the composition is heterogeneous and includes cardiac myocytes (orange), myofibroblasts/fibroblasts (green) and collagen fibers (purple). Depolarization of cardiomyocytes when an excitation propagates from left to right (brown arrows). Dotted arrows represent a conduction block, while dashed arrows represent slowed conduction. As a result of the zig-zag propagation of the wavefront (white arrows), the unipolar electrogram morphology is not symmetric, is prolonged and shows multiple deflections.

**FIGURE 5:**
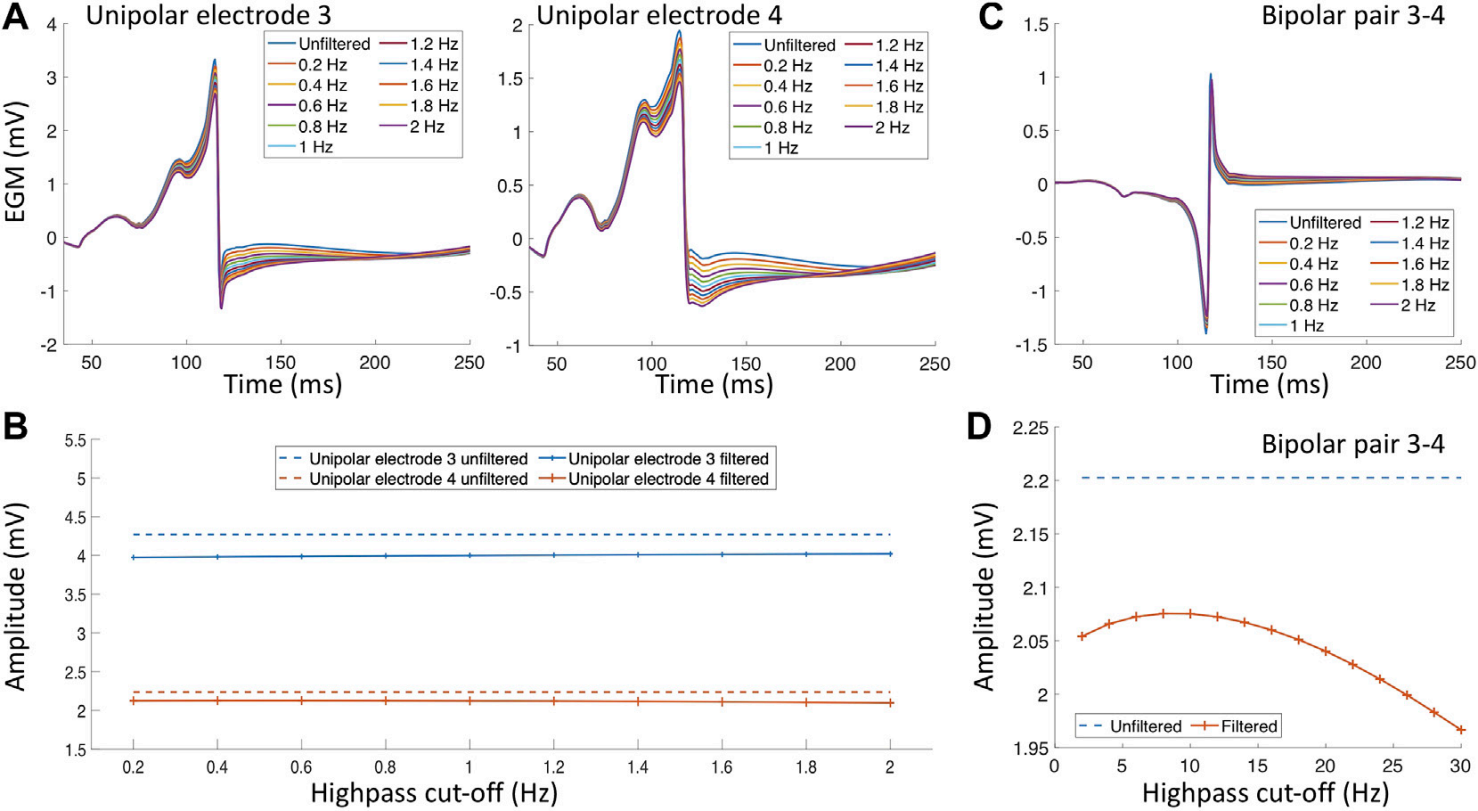
Filter effect on electrograms measured in the proximity of a fibrotic area. **(A)** Unipolar electrograms with different highpass filter values. **(B)** Effect of different highpass cut-off values on the unipolar electrogram amplitude. **(C)** Bipolar electrogram with different highpass cut-off values. **(D)** Effect of different highpass cut-off values on the bipolar electrogram amplitude.

Structural remodeling alters the cardiac substrate, and the depolarization wavefront often has to follow a zig-zag pattern (Figure 4 white arrows). The zig-zag pattern of the propagation is reflected in uEGM and biEGM as fractionation in the signal due to constantly changing orientation of the wavefront. Fractionation is defined as an increase of deflections, thus an increase in complexity of the signal as well as a prolongation of the EGM (Jacquemet and Henriquez, 2009; Verheule and Schotten, 2021). As previously mentioned, the highpass filter cut-off value affects the signal amplitude. In the presence of fibrotic tissue, uEGMs and biEGMs have a different frequency spectrum and are affected in a different manner. Figure 5D shows that there is no optimal cut-off frequency as previously reported by Starreveld et al. (2020). The filtered biEGM amplitude (orange line) drops due to the highpass cut-off but does not intersect the unfiltered amplitude (blue dashed line) as is the case for healthy myocardium (Figure 3D).

Many approaches have been proposed to model fibrotic cardiac tissue (Table 1) to understand the effect on the wavefront propagation and the corresponding electrograms (Ashihara et al., 2012; McDowell et al., 2013; Roney et al., 2016).

**TABLE 1:** Different modeling approaches to represent fibrotic tissue in computational models and their effect on simulated EGMs.

Modeling approach	Effect on EGMs	References
Myofibroblasts/fibroblasts coupled to myocytes	longer duration due conduction slowing in the fibrotic area	MacCannell et al. (2007), Ashihara et al. (2012), McDowell et al. (2013), Morgan et al. (2016), Roney et al. (2016), Zahid et al. (2016), Sánchez et al. (2019b)
Reduced conductivity in fibrotic region, potentially with gradient to surrounding tissue	peak-to-peak amplitude reduced and duration prolonged due to slow propagation of the wavefront	Krueger et al. (2014), Caixal et al. (2020), Lim et al. (2020), Beach et al. (2021)
Severely reduced conductivity in some elements in the fibrotic region	reduced peak-to-peak amplitude in the fibrotic area	Alonso and Bär, (2013), ten Tusscher and Panfilov, (2007), Clayton, (2018)
Removing some elements in the fibrotic region	fractionation and reduced peak-to-peak amplitude	Roney et al. (2016), Vigmond et al. (2016)
Edge splitting	fractionation depending on the length of the path	Jacquemet and Henriquez, (2009), McDowell et al. (2013), Mendonca Costa et al. (2014), Roney et al. (2016)
Reduction of conductivity in the transversal fiber direction	increased anisotropy of excitation propagation, effect on EGMs not yet studied	McDowell et al. (2012)
Reduction of conductivity in the transmural direction	excitation propagation dissociation between transmural layers, effect on EGMs not yet studied	Gharaviri et al. (2016), Irakoze and Jacquemet, (2020)

Creating a model of cardiac fibrotic tissue is not an easy task as fibrosis formation has been associated with different diseases (myocardial infarction (Liu et al., 2017), diabetes (Russo and Frangogiannis, 2016), autoimmune diseases (Tschöpe et al., 2021) and others), which produce different patterns of structural remodeling (interstitial, compact, diffuse, and patchy) (Nguyen et al., 2014). For example, it has been described that during an ischemic episode in the ventricle, the myocardium undergoes electrical remodeling (Mendonca Costa et al., 2018). From a macroscopic view, conduction velocity is reduced in the scar area, which can be modeled by decreasing the conductivity or by including isolating barriers (Balaban et al., 2018). Additionally, at a cellular scale the cardiac myocytes undergo electrical remodeling (Mendonca Costa et al., 2018). At the border zone of the ischemic area, cardiomyocytes lack oxygen which impacts their metabolism and increase acidity. This triggers a series of effects in the cell’s ion channels. To model these effects, the maximum conductance (Rodriguez et al., 2006; Loewe et al., 2018) of certain ionic channels are modified including an ATP-sensitive potassium channel (I_KATP_), which has a major contribution during ischemic episodes (Dutta et al., 2017).

**FIGURE 6:**
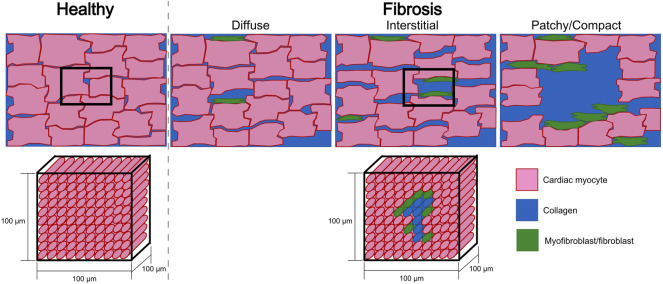
Sketches of a tissue cut for healthy and fibrotic tissue (top row). Fibrotic sketches represent different fibrotic patterns (diffuse, interstitial, patchy or compact). The bottom row depicts the homogenization assumption where a hexahedral mesh element of 100 μm × 100 μm × 100 μm represents several cardiac myocytes and has to assume one average set of properties that describes the electrophysiology of this group of cells. For fibrotic tissue, homogenization implies that one element contains different types of cells (cardiac myocytes and fibroblasts/myofibroblasts) and collagen. Also the electrophysiological characteristics of this piece of tissue has to be represented by one set of effective parameters.

Moreover, computational models of pathological tissue need to include fibrosis at the tissue scale. Fibrosis patterns (Figure 6) can be modeled using different approaches by assigning different properties to the mesh using for example, a random distribution (e.g., uniform or Gaussian) (Sánchez et al., 2019b; ten Tusscher and Panfilov, 2007; Alonso and Bär, 2013; Vigmond et al., 2016), by extracting the scar area from MRI (McDowell et al., 2012; Krueger et al., 2014; Morgan et al., 2016; Beach et al., 2021) or by using algorithms that synthetically generate similar patterns as observed in histological cuts of fibrotic tissue (Jakes et al., 2019; Pezzuto et al., 2019; Sutanto et al., 2020; Sánchez et al., 2021b).

Fibrosis can be modeled differently and many studies reduce the tissue conductivity such as for example, informed by microstructural modeling in Gokhale et al. (2017). The conductivity of the fibrotic areas can also be reduced in the transversal direction (McDowell et al., 2012) to represent lateralization of gap junctions, close to zero in all directions (Clayton, 2018) or affected by a no flux boundary condition (ten Tusscher and Panfilov, 2007; Alonso and Bär, 2013) to represent replacement fibrosis. The specific spatial distribution of conductivities or conduction velocity can be informed by fibrosis imaging such as the pixel intensity in late gadolinium enhanced magnetic resonance images (Krueger et al., 2014; Morgan et al., 2016; Caixal et al., 2020; Beach et al., 2021) or using a mathematical function determined from EGM amplitude (Lim et al., 2020). Within the regions, either uniform conductivities can be assumed or a gradient from the center of the fibrotic area to the healthy surrounding tissue is assumed.

Furthermore, the edge splitting method has been proposed to separate the computational mesh along its edges with the aim to reproduce the effect of collagen deposition in fibrotic tissue that separates the cardiac myocytes (Mendonca Costa et al., 2014). Edge splitting consists of splitting the nodes along and edge to disconnect adjacent elements creating an alternative path for the wavefront propagation in the cardiac tissue. However, reducing the conductivity or splitting the edges of the mesh does not capture the effect of increased cellular heterogeneity in the cardiac tissue (fibroblast-myocyte coupling) and the inflammatory response. To model cellular heterogeneity, myofibroblast or fibroblast models have been introduced (MacCannell et al., 2007; Ashihara et al., 2012; Morgan et al., 2016; Roney et al., 2016; Sánchez et al., 2021b). Myofibroblasts or fibroblasts were electrically connected to the myocytes by gap junctions. There are equivocal data about the exact conductance of these gap junctions and the number of fibroblasts that a myocyte couples to. In computational models, the value of conductance ranges between 0.5 nS to 2 nS and up to 9 fibroblasts are considered Morgan et al. (2016), MacCannell et al. (2007), Maleckar et al. (2009), Rook et al. (1992), Sánchez et al. (2019a), Seemann et al. (2017). The inflammatory response (myocyte-fibroblast paracrine interactions) has been modeled by altering the maximum conductance of the sodium ion channel (reduced by 50%), the maximum conductance of the L-type calcium ion channel (reduced by 50%), and the maximum conductance of the inward potassium rectifier ion channel (reduced by 40%) (Zahid et al., 2016), as reported by *in vitro* experiments (Avila et al., 2007; Ramos-Mondragón et al., 2011).

Lately, Vigmond et al. (2016) proposed to represent fibrotic tissue in a monodomain model by removing the elements of the mesh to capture the effect of the low conductive extracellular medium and the absence of an intracellular current path. One advantage of the proposed modeling approach, is that there is no flux of current towards the fibrotic tissue; therefore, there are no source elements that will contribute to the calculated extracellular potential. Using this modeling approach, the authors observed that at the percolation threshold (Alonso and Bär, 2013) the fibrotic tissue was be able to trigger and maintain an arrhythmia. The EGMs calculated over the fibrotic tissue exhibit fractionation due to the zig-zag patterns of depolarization in the cardiac tissue in this modeling approach. Moreover, the study also looked at the impact of the mesh resolution when modeling fibrotic tissue and showed that in meshes with a resolution of 300 μm conduction block was reached at lower degrees of fibrosis than in meshes with finer resolution ( < 100 μm).

Using a realistic geometry Jacquemet et al. (2003) studied the morphology of uEGMs during different atrial fibrillation propagation patterns. The authors showed that different propagation patterns (plane waves, spiral waves, and wavefront collision) lead to different uEGM morphology (symmetry and amplitude) and that asymmetric signals (Figure 2B) occurred in less than 2% of the cases in homogeneous substrate. However, the increase of heterogeneities in the cardiac tissue also increases the asymmetry and reduces the amplitude of the EGM(van der Does and de Groot, 2017). Frontera et al. (2018) showed how different depolarization patterns affected the biEGM morphology. High peak-to-peak amplitude and short duration of biEGMs are wavefront collisions or pivotal points, low peak-to-peak amplitude and EGM prolongation are associated with slow conduction areas. The authors remarked how understanding the genesis of the electrograms is a key factor to improving the arrhythmia treatments.

**FIGURE 7:**
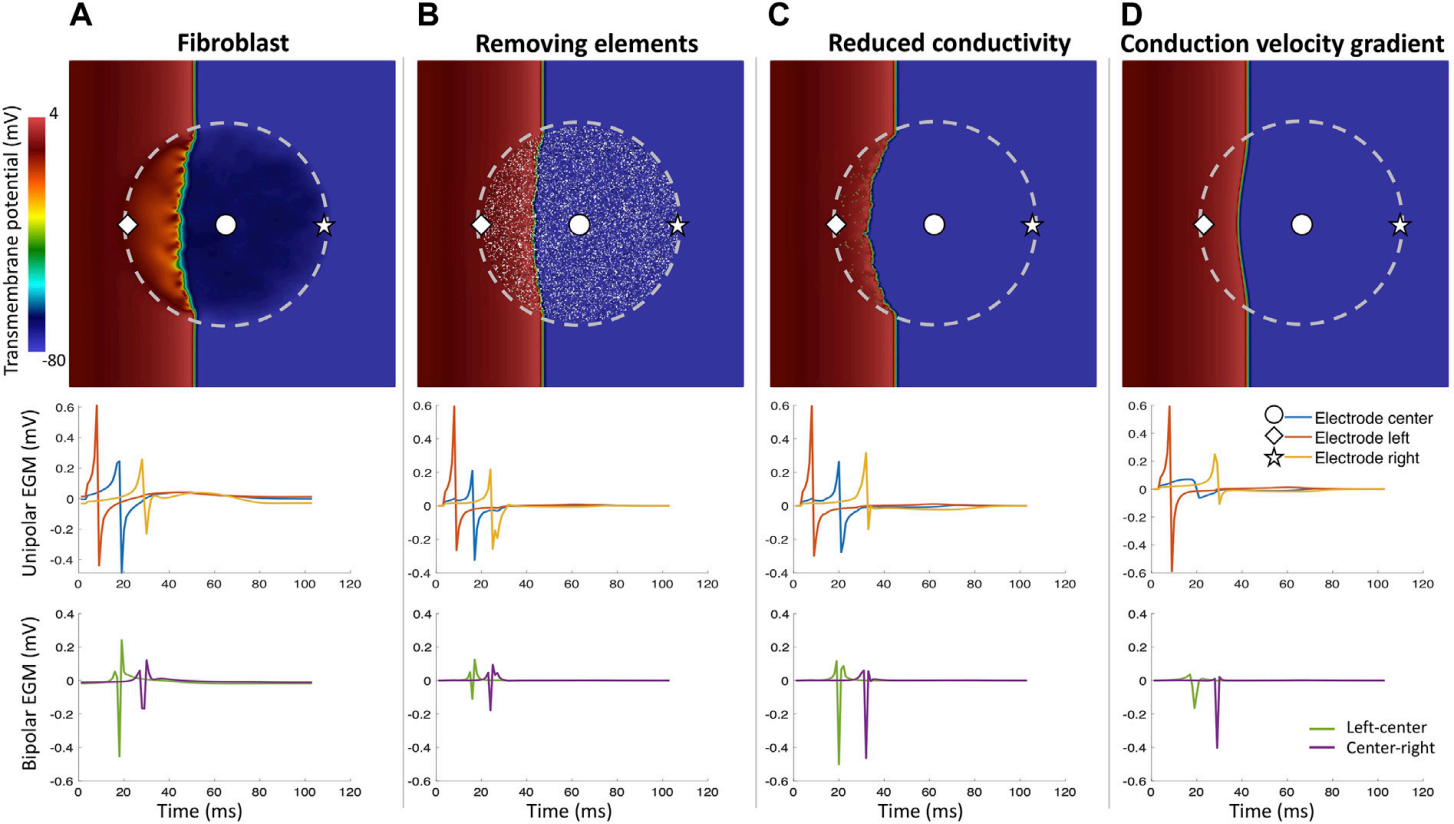
Different fibrosis modeling methodologies and their corresponding unipolar and bipolar electrograms. Central fibrotic area (dashed line), the choice of the modeling approach affects the resulting uEGMs and biEGMs. **(A)** fibroblasts coupled to myocytes in the fibrotic region; **(B)** removing a share of elements in the fibrotic region from the computational domain; **(C)** severely reducing conductivity in a share of elements in the fibrotic region but the spatial transmembrane voltage gradients of fibrotic elements still contribute to the EGMs; **(D)** conductivity gradient from center of the fibrotic region to the surrounding healthy tissue. Absolute EGM amplitudes are smaller than *in vivo* due to the small size of the tissue patch.

Including heterogeneous tissue composition in the model changes the wavefront propagation in the cardiac tissue (McDowell et al., 2012; Campos et al., 2013; Mendonca Costa et al., 2014; Roney et al., 2016) (Figure 7). Roney et al. (2016) showed how different modeling approaches of cardiac fibrosis can change the propagation in the cardiac tissue and affect the morphology of EGMs. In that study, Roney et al. (2016) modeled fibrosis as conduction disturbances (lower conductivity, edge splitting, or removing elements). They included electrical remodeling of the cardiac myocyte due to inflammatory processes mediated by transforming growth factor-*β*1, myocyte-fibroblast coupling and combinations of the preceding. EGM morphology was mostly affected when fibrosis was modeled by edge splitting or removing the elements (Figure 7) as also shown previously. In addition, including fibroblast coupling has an organizing effect on rotor dynamics, also shown by other studies (McDowell et al., 2012; Sánchez et al., 2019b).

The amplitude of the EGMs can also be affected by conduction impairment along certain axes (McDowell et al., 2012; Gharaviri et al., 2016; Irakoze and Jacquemet, 2020). Gharaviri et al. (2016) created a model of the cardiac tissue that enables the study of dissociation between transmural layers, for example, dissociation between the subendocardial and the subepicardial myocardium as can be caused by endomysial fibrosis. Moreover, Saba et al. (2009) described how the epicardial EGM amplitude varies in the ventricle with the thickness of the epicardial fat layer. The authors showed that biEGM amplitude was inversely related to epicardial fat thickness. Thus, using a voltage cut-off of 0.5 mV to define scar tissue would lead to identifying also healthy areas with overlaying fat and more information needs to be used to define epicardial tissue characteristics.

## 5 Other Factors Impacting Intracardiac Signals

EGM morphology and amplitude are also affected by electrode polarization, excessive contact pressure, catheter motion (Oesterlein et al., 2016), electromagnetic interference (Unger et al., 2019), near field and far field effects (Schicketanz et al., 2021), and poor grounding. However, most *in silico* experiments do not consider these factors, which might alter the EGM characteristics. Simulation studies have created a model of clinical noise which covers the electromagnetic interference (Sánchez et al., 2021a; Nothstein et al., 2021). However, further aspects likely need to be considered explicitly if their influence is relevant for the intended use of the model.

## 6 Research Gaps and Potential Future Developments

Modeling of the cardiac tissue has significantly advanced understanding of the electrical propagation and the measured intracardiac EGMs. There is consensus on how to assign the properties of the computational model to represent healthy myocardium and the advantages and limitations of the different approaches to compute the extracellular potentials are mostly characterized. However, the question how to model fibrosis is far from being ultimately answered and will most likely continue to depend on the question of interest to be answered with a specific model. Additionally, the mesh resolution used in most of the studies of ≈300 μm determines the degree of homogenization (Figure 6). Spatial discretization of the mesh at the cellular level should be considered to study the influence of microstructural heterogeneity in the tissue (e.g., fibrosis) on EGMs (Figure 4). In addition, such models with subcellular resolution would enable to investigate to which degree discontinuous propagation within a cell vs. between cells leads to fractionation in healthy tissue. Here, we presented an overview of the commonly used methods and their corresponding EGMs.

Over the last years, the human cardiac digital twin has been under development to suggest personalized treatments for cardiac arrhythmias. Gillette et al. (2021) proposed an automated framework to generate a patient’s digital twin from clinical data and Nagel et al. (2021) proposed a statistical approach to generate a population of anatomical models. While the anatomical model can be accurately generated from magnetic resonance images or statistical shape models, functional twinning can be achieved by tuning a phenomenological model or using generalized global properties for the cardiac tissue. Functional information will impact the morphology and amplitude of the EGM. However, over the years, different studies proposed distinct methodologies to extract structural and functional information from the EGM signals. One open question is still the possibility of obtaining repolarization times from EGMs as repolarization of the cardiac tissue plays a pivotal role for the initiation of arrhythmias (Rivaud et al., 2021). From simulations of atrial electrophysiology, Celotto et al. (2021) proposed a method to detect areas of parasympathetic innervation from the amplitude of the repolarization EGM. Verrier et al. (2016) showed that repolarization times can be recovered from EGMs for both the atrium and the ventricle in a controlled clinical environment. However, initial experience in other groups including our own suggest that reliably obtaining atrial repolarization information from EGMs remains a challenge.

Different studies demonstrated discrepancies when using the same voltage threshold (for example, 0.5 mV) to distinguish healthy from pathological tissue when mapping during different rhythms (sinus rhythm and AF) (Rodríguez-Mañero et al., 2018; Nairn et al., 2020b; Nairn et al., 2022). Nairn et al. (2022) looked at how the amplitude of EGMs changed when electroanatomical mapping was performed under three different rhythms (sinus rhythm, native AF, and induced AF). The authors proposed not only one single cut-off voltage value for the entire atrium but regional voltage thresholds to minimize the discrepancies between different mapping rhythms. Computer models could help to further characterize the voltage relations during different rhythms and to overcome the use of a voltage threshold to distinguish the cardiac substrate (healthy and fibrotic) by combining *in vivo* data and *in silico* data to fully exploit the information contained in EGMs (Sánchez et al., 2021a). Additionally, computer models of cardiac electrophysiology could aid the design of medical devices helping in understanding the factors that affect EGMs to raise awareness for them (Oesterlein et al., 2016; Pollnow et al., 2017; Beheshti et al., 2018; Hwang et al., 2019) as well as to inform the choice of parameters to improve the technologies as proposed for cardiac resynchronization therapy (Jolley et al., 2010).

Understanding the functional relationship between the discrete structure and continuum behaviour of cardiac tissue at microscopic and macroscopic levels is a significant challenge (Gokhale et al., 2017). At the microscopic level, Tveito et al. (2017) and Bécue et al. (2017) proposed a cell-by-cell approach that explicitly models the extracellular, membrane and intracellular domain. However, cell-by-cell models are computationally expensive and will require an increase of computational resources such that finer meshes up to cellular resolution can be handled efficiently (Potse et al., 2020). At the macroscopic level, reduced order models (Fresca et al., 2020) could help to reproduce in detail the electrophysiology of the cardiac tissue without losing important details that will determine the vulnerability of the tissue to arrhythmia. Recently, (Herrero Martin et al., 2022) explored the use of Physics Informed Neural Networks (PINN) to model the electrical propagation in the cardiac tissue. The authors introduced electrophysiology models to the neural network and were able to reconstruct the spatial-temporal dynamics of the action potential and its propagation. One of the big drawbacks of these approaches is the amount of data needed to train the network in order to predict different possible propagations patterns.

Software plays a fundamental role in cardiac modeling. Recent work demonstrated significant speedup of simulations of cardiac electrophysiology (Sundnes et al., 2006; Seemann et al., 2010; Cooper et al., 2015; Quarteroni et al., 2017; Sánchez et al., 2020; Plank et al., 2021). However, it remains to be seen how effectively GPUs can be integrated into large-scale cardiac simulations. Regardless, several numerical libraries are currently available, opening the door to accelerate cardiac electrophysiology simulations (Anzt et al., 2020; Mills et al., 2021).

## 7 Conclusion

Models of cardiac tissue electrophysiology have played an essential role in advancing our understanding of action potential propagation in the heart and the genesis of EGMs. Despite the significant progress of different modeling approaches and efficient numerical software, there are substantial challenges, such as modeling of the microstructure at a close-to-cellular scale, modeling the different aspects of fibrosis, electrophysiological heterogeneity as well as realistic electrode configurations. Dedicated simulation studies with refined models will help to further elucidate the different factors that contribute to EGM genesis and impact their morphology.
