# Corrigendum: A review of healthy and fibrotic myocardium microstructure modeling and corresponding intracardiac electrograms

**DOI:** 10.3389/fphys.2024.1477339

**Published:** 2024-09-05

**Authors:** Jorge Sánchez, Axel Loewe

**Affiliations:** Institute of Biomedical Engineering, Karlsruhe Institute of Technology (KIT), Karlsruhe, Germany

**Keywords:** cardiac modeling, fibrosis, electrogram, multiscale, microstructure

In the published article, there was an error in Figure 1 as published. The colors that represent the extracellular field did not represent the correct direction of the field**.** The corrected Figure 1 and its caption appear below.

**FIGURE 1:**
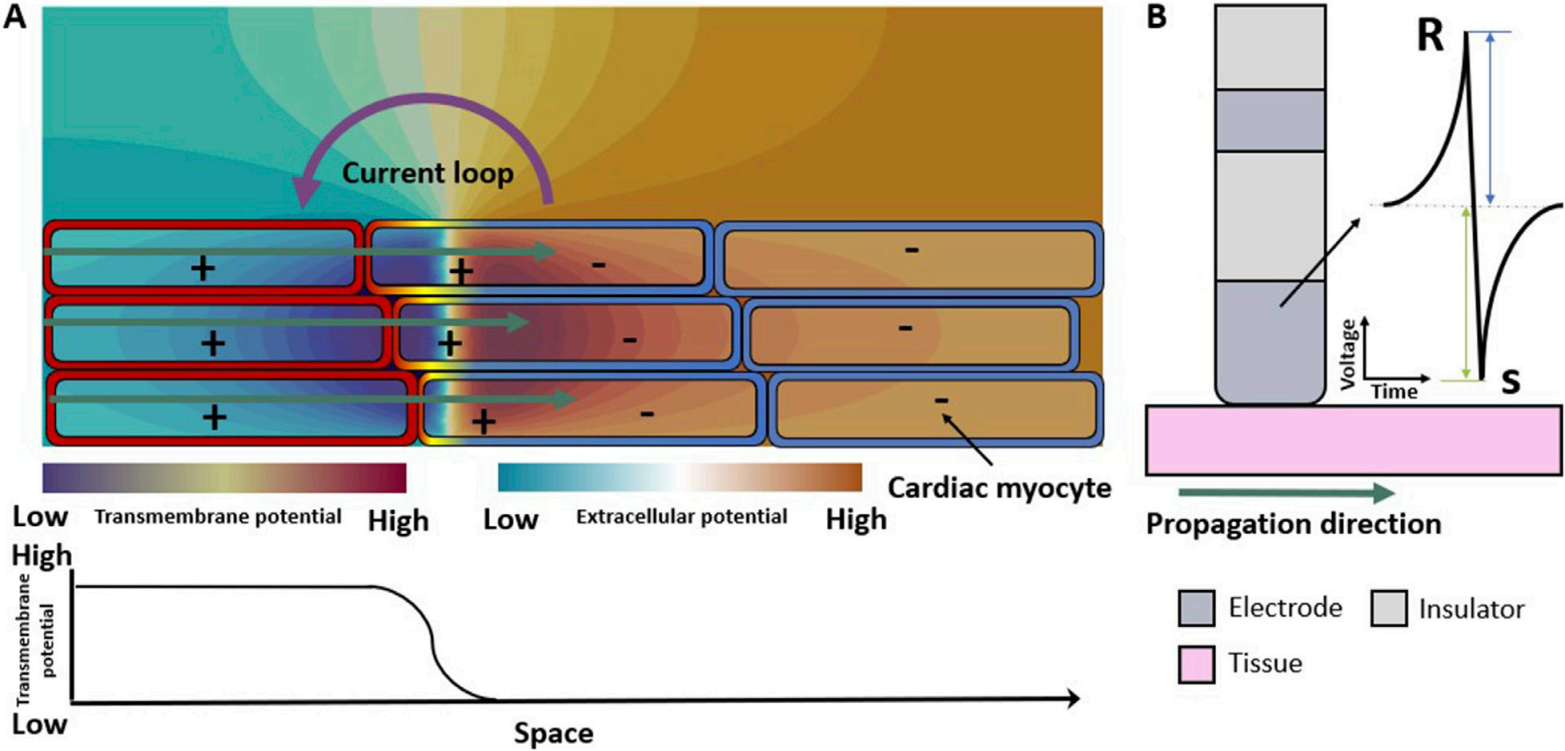
Electrical propagation in healthy cardiac tissue. **(A)** Extracellular field caused by the depolarization of cardiomyocytes when an excitation propagates from left to right (green arrows). Spatial transmembrane voltage distribution is color-coded on the membranes and shown in the bottom row. The leftmost cells are already depolarized an in the action potential plateau while those on the right are still at resting membrane voltage. **(B)** Symmetric unipolar electrogram measured at the surface of the cardiac tissue (pink). The initial positive wave (R-peak) is caused by the wavefront approaching the electrode (dark gray), the polarity changes when the wavefront passes underneath the electrode, and the S-peak is caused by the wavefront traveling away from the measuring electrode.

The authors apologize for this error and state that this does not change the scientific conclusions of the article in any way. The original article has been updated.

